# Development and initial validation of a modified lymphocyte transformation test (LTT) assay in patients with DRESS and AGEP

**DOI:** 10.1186/s13223-022-00729-4

**Published:** 2022-10-09

**Authors:** Chris Weir, Jamma Li, Richard Fulton, Suran L. Fernando

**Affiliations:** 1 Northern Blood Research Laboratory, Kolling Institute of Medical Research, Royal North Shore Hospital and the Sydney Medical School, University of Sydney, Sydney, NSW Australia; 2 Department of Clinical Immunology and Allergy, Royal North Shore Hospital, Sydney, NSW Australia; 3 Immunology Laboratory, New South Wales Health Pathology, Royal North Shore Hospital, Level 11, Kolling Building, St Leonards, Sydney, NSW 2065 Australia

**Keywords:** DRESS, LTT, AGEP

## Abstract

**Background:**

The lymphocyte transformation test (LTT) is an in vitro assay used to diagnose drug induced hypersensitivity reactions by detecting the activation and expansion of drug-specific memory T cells to the suspected implicated drug. Traditionally radiolabelled thymidine (3H-thymidine) has been used but requires the handling and disposal of radioactive materials.

**Objective:**

To examine safe alternatives to 3H-thymidine, test assay modifications for improved assay sensitivity and evaluate the modified LTT in patients with DRESS and AGEP.

**Methods:**

Four proliferation detection assays (BRDU, CyQUANT™, MTT and XTT) were screened for LTT sensitivity. XTT the most sensitive and practical was selected for further evaluation Modifications like autologous serum (AS) and regulatory T cell depletion (T-REG) were tested for improved assay sensitivity. Finally, an initial evaluation of the XTT–LTT was performed in 8 patients with DRESS and 2 with AGEP including cytokine testing.

**Results:**

Of the non-radioactive alternatives we tested, XTT a colorimetric assay was the most sensitive and practical to move to evaluation. The addition of AS increased background signal. Depletion of T-REGs improved sensitivity but cell sorting time and risk of contamination limited benefit.

Of eight patients diagnosed with DRESS and 2 with AGEP tested with XTT–LTT assay results showed our assay matched clinical findings of implicated drugs in 8/10 patients when using a stimulation index (SI) ≥ 2 and 8/10 with analysis by ANOVA. All ten patients were correctly diagnosed by either analysis.

**Conclusion:**

XTT appears to be a safe, viable alternative to 3H-thymidine, with high sensitivity and allowing direct cytokine quantification on specific patient cells.

## Introduction

Type 4 hypersensitivity reactions are T cell-mediated reactions that occur in response to contact with certain allergens, including drug allergens. These reactions include severe cutaneous adverse reactions (SCAR) which are life-threatening. This paper focusses on two types of SCAR, drug reaction with eosinophilia and systemic symptoms (DRESS) and acute generalised exanthematous pustulosis (AGEP).

DRESS is characterised by fever, rash, lymphadenopathy, leucocyte abnormalities including eosinophilia, and internal organ involvement, usually from 3 weeks of exposure to the drug culprit. Drugs and metabolites bind directly to human leucocyte antigen (HLA) loci through the “p–i” concept (pharmacological interaction of drugs with immune receptor), with specific HLA variants carrying increased risk of DRESS for different drug culprits. Reactivation of human herpes viridae family viruses are hypothesised to dysregulate T cell pathways, leading to T cell activation and increased antiviral response with production of TNFα, IL-2, and IFN-γ. Expansion of regulatory T cells also occurs in the acute phase of DRESS [1].

The resulting eosinophilic infiltration of organs with subsequent damage occurs in response to chemokines including eotaxin-1 and TARC, in synergy with IL-5 produced from T cells [2].

AGEP is caused by a drug culprit in > 90% of cases. It is characterised by fever, neutrophilia, and appearance of disseminated sterile pustules within 2 days of exposure. The pathogenesis is unclear but involves activation and proliferation of drug-specific T cells. It is associated with increased levels of chemokine CXCL8, IL-17, IL-22 and GM-CSF.

Identifying the drug culprit is necessary to prevent future reaction. This is challenging when there is more than one implicated drug. In vivo and in vitro tests are available for identification of drug culprits. Patch testing has at best, a sensitivity of 30–60% for DRESS, and 18–58% for AGEP depending on the drug culprit [3–5]. Lymphocyte transformation tests (LTT) measures proliferation of lymphocytes to a drug in vitro*.* Existing LTT shows high specificity (98–99%) but lower sensitivity in DRESS patients (73%) [6]. The interferon-gamma (IFN-γ)-ELISpot was found in the same review to have a similar sensitivity and specificity in DRESS-patients [7]. Small numbers published for LTT in AGEP suggest high sensitivity and specificity [7].

Classic LTT relies on incorporation of 3H-thymidine in its method, and laboratories are progressively effacing techniques using radioactivity due to safety the need for specialised disposal and cost. Our group aimed to develop and optimise a non-radioactive method of LTT for identifying drug culprits in DRESS and AGEP. We compare several non-radiolabelled proliferation detection assays in the traditional 96 well assay format and select the optimal assay for method validation in DRESS and AGEP patients.

## Methods

### Study outline

Ethical approval was obtained from RNSH human ethics committee (RESP/16/255). Following provision of information on the study, DRESS and AGEP patients diagnosed according to the RegiSCAR criteria gave informed written consent to have their blood collected for the study. For initial assay optimisation, blood was collected from healthy volunteers prior to being validated in DRESS and AGEP patients.

### Blood collection and PBMC isolation

Fresh heparinised blood was collected for all experiments and PBMCs were collected using our previously published method [8]. After collection off interface, PBMCs were washed with PBS and counted using a haemocytometer. Nine out of ten patients were in the recovery phase at time of blood collection and testing. Patient 7 was on low dose prednisone (under 10 mg) at the time of testing.

### Proliferation assay evaluation

After PBMC collection and counting, cells were added to wells of a 96 well plate at a concentration of 200,000 cells per well in triplicate. Phytohaemagglutinin (PHA) at 5 µg/ml (Sigma) and T-Cell Transact ™ beads (Miltenyi Biotec) at a 1:200 dilution, were used as positive controls. Negative control wells contained cells in culture media alone (RPMI + 10% FCS). Samples were incubated 37 °C/5% CO_2_ for 6 days to allow for cell-stimulant interaction. At day 6, bromodeoxyuridine (BRDU), CyQUANT™, tetrazolium salt (3-(4,5-dimethylthiazol-2-yl)-2,5-diphenyltetrazolium bromide (MTT) and tetrazolium salt (sodium 3′-[1- (phenylaminocarbonyl)- 3,4- tetrazolium]-bis (4-methoxy6-nitro) benzene sulfonic acid hydrate (XTT) proliferation detection assays were evaluated to determine which was most sensitive for determining cell proliferation in a non-radiolabelled LTT assay.

Briefly BRDU detection was performed using the Merck/Millipore Cell Proliferation ELISA, BrdU (colorimetric) assay (Catalogue number 2752) according to the manufacturer’s instructions. The CyQUANT™ NF cell proliferation assay kit (C35006) was performed as per manufacturer’s protocol. MTT (Sigma/Merck, Catalogue number M2128) at a stock concentration of 5 mg/ml was added to each well for a final concentration of 0.5 mg/ml (20 µl per well) and incubated for 4 h at 37 °C. As T cells are not adherent, plates were spun at 1400 rpm for 10 min and supernatants carefully removed before addition of DMSO as a solvent to dissolve formazan crystals in the cells and plate read at 590 nm. Absorbance was then read at 570 nm. For XTT detection the (Roche/Sigma) cell proliferation kit II (XTT) was performed directly as per manufacturer’s instructions and read at 4, 6 and 24 h at 492 and 690 nm.

### Other modifications tested

Once XTT had been shown to be the most sensitive and practical of the proliferation detection methods, other modifications to the assay were tested. The addition of autologous serum (AS) was tested to see the effect of proliferation on PHA and CD3 + /CD28 + stimulated cells. AS is used in LTT assays particularly for Nickel allergies and reported to improve sensitivity. PBMCs were collected from three healthy controls. Briefly, RPMI media with 10% FCS was compared to RPMI with 10% FCS + 5% AS, or RPMI + 5% AS on PHA and CD3 + /CD28 + stimulated cells over 6 days.

Regulatory T cell (T-reg) depletion by flow cytometric sorting was also tested. T-Regs may supress T cell, activation so depletion was tested to see if this improved the XTT–LTT assays sensitivity. Purified PBMCs from three healthy controls were stained with an anti-CD25 antibody (BD Biosciences) for 15 min at room temperature before CD25 + positive and negative PBMC’s were sorted using a BD FACSAria™. CD25 + positive and negative cell populations were spun at 1400 rpm for 5 min and washed in PBS prior to being counted and plated at 100,000 to 200,000 cells per well for testing in LTT assay. T-reg depleted (sorted) and unsorted PBMC’s ± 5% AS were compared after stimulation with PHA and CD3 + /CD28 + beads for 6 days. Positively sorted CD25 + ve T-regs were also tested for proliferation levels to PHA and CD3 + /CD28 + stimulation after 6 days with and without the addition of 5% AS.

### Patient LTT

After XTT-modified LTT (XTT–LTT) assay optimisation, 8 patients diagnosed with DRESS and 2 with AGEP were tested with the XTT–LTT against implicated drugs and metabolites in some cases. A further 6 age and gender matched controls for patients 2,3,4,5,7 and 8 with no history of reactivity to any matched implicated drug were also added to the study. These drugs included vancomycin, piperacillin/tazobactam (Tazocin), sulfasalazine, allopurinol, apixaban, sulfapyridine, sulfamethoxazole, sulfamethoxazole + trimethoprim, amoxicillin/clavulanic acid, ceftriaxone, ceftotaxime, cefazolin, ceftazidime, benzylpenicillin, amoxicillin. All drugs were tested at 100 µg/ml, 50 µg/ml, 25 µg/ml, 12.5 µg/ml, 6.25 µg/ml and 3.1 µg/ml in duplicate or triplicate depending on the amount of patient cells available. The drugs allopurinol, oxypurinol, sulfapyridine and sulfamethoxazole had to be solubilized in DMSO prior to testing. PHA (Sigma) 5 µg/ml and T-Cell Transact ™ beads (Miltenyi Biotec) at a 1:200 dilution, were used as positive controls. Negative control wells contained cells in RPMI media + 10% FCS alone. Drug-Cell interaction was carried out for 6 days at 37 °C/5% CO_2._ At Day 6, 50 µl of XTT solution was added to each well and read at 492 and 690 nm at 4, 6 and 24 h. All plates were frozen post 24 h read at − 80 °C for future cytokine analysis.

### Cytokine analysis

Ninety-six well plates stored at − 80 °C were removed and allowed to thaw at room temperature. Implicated drug-positive wells for each patient, along with PHA, T-Cell Transact ™ bead positive controls and negative controls were screened for interleukin-2 (IL-2), interleukin-4 (IL-4) and interleukin-5 (IL-5) levels by ELISA according to the manufacturers’ instructions (R and D Systems).

### Data analysis by one way ANOVA and stimulation index (SI)

#### One-way ANOVA

Data collected from each XTT–LTT patient or control were analysed using one-way ANOVA’ with Tukey’s multiple comparison post-test. All data was analysed with Prism 5.0 (Graph pad, La Jolla, CA).

#### Stimulation Index (SI) calculation

Stimulation Index (SI) was calculated by dividing the XTT signal (492–690 nm) of drug stimulated samples with the XTT signal of unstimulated samples with no drug added (negative control). A SI value of ≥ 2 was considered a positive result.

## Results

### Evaluation of proliferation detection assays BRDU, CyQUANT™, MTT and XTT

Four different proliferation assays BRDU, CyQUANT™ NF, MTT and XTT were analyzed for their potential effectiveness for inclusion into LTT. BRDU, initially assessed in an ELISA format, produced a no or weak signal due to failure of the fixation step. Extending the fixation time slightly improves the signal but the PHA and CD3 + /CD28 + bead controls are not significant by ANOVA and fails to achieve a SI (Table 1). The CyQUANT™ produces a slightly better PHA and CD3 + /CD28 + signal but the SI is < 1.2 even at 3 h reading. The MTT is sensitive for use in LTT but is limited in performance to a single time point and the need for the addition of DMSO to dissolve formazan crystals. The XTT detects the strongest PHA and CD3 + /CD28 + signal of all assays, the signal is read at multiple time points and is the easiest to perform Table 1 summarises the results from this comparative assessment of the four proliferation assays.

**Table 1:** Results of assessment of all proliferation assays tested as a potential detection method for LTT

ASSAY	Multiple time point read outs	Positive signal strength		Supernatant for cytokine analysis	Comments
		PHA	Transact beads		
BRDU	No, single	Neg SI [1] ANOVA (NS)	Weak (SI) 1.2 < ANOVA (NS)	Taken off before assay	Required spinning cells down to prior to fixation. Fixing step failed possibly due to T cells being small and non-adherent. Low sensitivity
CyQUANT™ NF	Yes	Weak SI < 1.2 ANOVA (NS)	Weak SI < 1.3 ANOVA (NS)	Taken off before assay	Required spinning cells down and removing supernatant. Signal weak after 60 min slight increase at 3 h
MTT	No, single	High SI 2.4 ANOVA *p < 0.05	High SI 2.6 ANOVA *p < 0.05	Taken off before assay	Assay sensitive enough but can only be read at one time point. Requires addition of DMSO for reading—thus no further analysis could be performed
XTT	Yes	High SI 2.8 ANOVA **p < 0.01	High SI 2.9 ANOVA **p < 0.01	Taken before or after assay	Simple addition of XTT Can be read at multiple time points (4, 6 and 24 h). Plates can be frozen after reading for further analysis

All future experiments were performed using XTT detection

*NS* Not significant, *NEG* Negative, *BRDU* bromodeoxyuridine, *CyQUANT™* CyQUANT NF cell proliferation assay kit, *MTT* tetrazolium salt (3-(4,5-dimethylthiazol-2-yl)-2,5-diphenyltetrazolium bromide, *XTT* tetrazolium salt (sodium 3´-[1- (phenylaminocarbonyl)- 3,4- tetrazolium]-bis (4-methoxy6-nitro) benzene sulfonic acid hydrate

SI greater > 2 positive, ANOVA *p < 0.05, **p < 0.01

### Analysis of T-Reg depletion and addition of 5% autologous serum

**Fig. 1:**
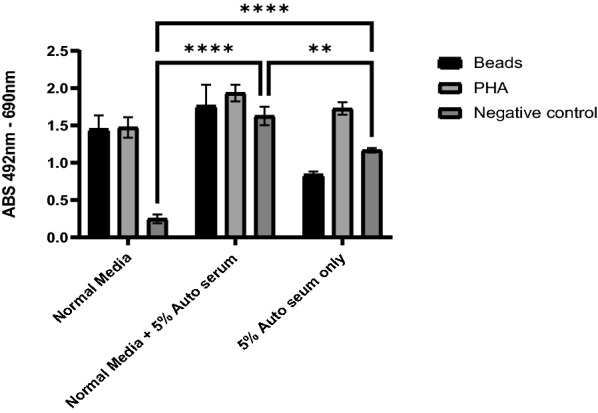
shows the effect of addition of 5% autologous serum to normal media with 10% FCS, and media without FCS. The addition of 5% serum either alone or into normal media significantly increased the level of signal from the negative control

Variations of the traditional 3H-thymidine LTT assay included the addition of 5% autologous patient serum. The results in Fig. 1 demonstrate that the addition of autologous serum significantly increased the negative control signal. When added to media without FCS, a similar trend occurred but with a decrease in signal from CD3 + /CD28 + stimulated T cells.

**Fig. 2:**
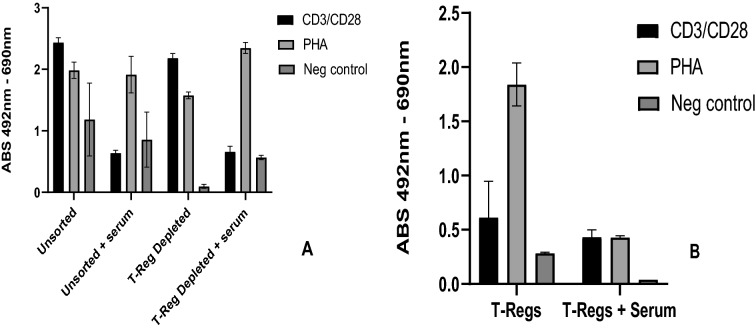
**A** Compares unsorted PBMC’s ± 5% autologous serum v T-Reg depleted PBMC’s ± 5% autologous serum. **B** Effect of 5% autologous serum on T-Reg stimulation ± 5% autologous serum

Depletion of CD25 + T-regs was tested against non-depleted PBMCs to ascertain if their depletion increases the signal to PHA and CD3 + /CD28 + beads. Figure 2A demonstrates that depletion of T-regs did not improve PHA or CD3 + /CD28 + stimulation but did decrease signal of the negative control. Addition of 5% autologous serum decreased CD3 + /CD28 + stimulation and increased the signal of the negative control. A similar effect was seen when 5% autologous serum was added to T-regs themselves (Fig. 2B). The duration of sorting-time for these assays was approximately one hour for a few million cells and was dependent on amount of PBMCs initially collected. Contamination during the sorting process resulted in bacterial growth in culture for 1 of 3 sorting experiments and thus had to be discarded.

### Patient diagnosis and histories

The clinical details of patients enrolled for the validation are detailed in Table 2.

**Table 2:** Clinical characteristics of patients enrolled in the study

	Age at Diagnosis, years	Gender (M/F)	Implicated drug	Diagnosis	Time from first dose to onset of first symptom, days	Time from first dose to onset of rash, days	Clinical characteristics	Skin biopsy result	Treatment	Time to resolution after treatment commenced, days
P1	42	F	Vancomycin	DRESS	11	12	Fever Eosinophilia 2.1 × 10^9^/L Lymphocytosis 6.0 × 10^9^/L Rash > 50% BSA Hepatitis	Variable epidermal spongiosis Superficial dermal perivascular lymphohistiocytic infiltrate with scattered perivascular eosinophils	Ceased implicated drug Corticosteroids IVIG	22
P2	77	M	Piperacillin/ Tazobactam (Tazocin)	DRESS	25	25	Fever Eosinophilia 1.0 × 10^9^/L Rash > 50% BSA Hepatitis	Lichenoid inflammation in epidermis Mild perivascular lymphocytic infiltrate with occasional eosinophils in dermis	Ceased implicated drug Corticosteroids	21
P3	21	F	Sulfasalazine	DRESS	26	26	Fever Lymphadenopathy – cervical and inguinal Eosinophilia 3.3 × 10^9^/L Lymphocytosis 9.0 × 10^9^/L Rash > 50% BSA Hepatitis	Lichenoid inflammation in epidermis Lymphohistiocytic infiltrate in dermis is predominantly perivascular but also at dermatoepidermal junction with isolated eosinophil	Ceased implicated drug Corticosteroids	36
P4	43	F	Allopurinol	DRESS	17	25	Fever Eosinophilia 2.7 × 10^9^/L Lymphocytosis 4.2 × 10^9^/L Rash > 50% BSA Hepatitis	Mild to moderate infiltrate in dermis predominantly perivascular with lymphocytes and eosinophils	Ceased implicated drug Corticosteroids	20
P5	18	M	Sulfasalazine	DRESS	35	37	Fever Lymphadenopathy —occipital, cervical, axillary, inguinal Eosinophilia, value unavailable Rash > 50% BSA Hepatitis	Not performed	Ceased implicated drug Corticosteroids	60
P6	54	F	Apixaban	AGEP	1	1	Fever Neutrophilia 13.9 × 10^9^/L Pustular rash	Not performed	Cease implicated drug Topical corticosteroids	7
P7	53	M	Tazocin	DRESS	28	28	Fever Eosinophilia 6.5 × 10^9^/L Rash > 50% BSA Hepatitis	Epidermis—mild spongiosis with foci of lymphocyte exocytosis Superficial dermis—perivascular lymphocytes and histiocytes, and occasional eosinophils	Cease implicated drug Corticosteroids IVIG	20
P8		F	Amoxicillin + clavulanic acid	DRESS			Rash > 50% BSA Hepatitis Acute kidney injury	Epidermis—mild spongiosis Prominent papillary dermal oedema, dermal perivascular and interstitial infiltrate with lymphocytes, histiocytes and occasional eosinophils, neutrophils and plasma cells	Cease implicated drug Corticosteroids	10
P9	62	F	Lenalidomide	DRESS	28	28	Fever Eosinophilia 5.0 × 10^9^/L Rash > 50% BSA Conjunctivitis Hepatitis Acute kidney injury	Epidermis—mild spongiosis with foci of lymphocyte exocytosis Dermis—patchy perivascular lymphocytic infiltrate with occasional eosinophils	Cease implicated drug Corticosteroids	17
P10	66	M	Benzylpenicillin	AGEP	1	1	Pustular rash Neutrophilia 27 × 10^9^/L	Epidermis—spongiosis, psoriasiform hyperplasia and scattered intraepithelial lymphocytes, small subcorneal pustules, and spongiotic vesicles with degenerating inflammatory cells Dermis—markedly oedematous, prominent perivascular inflammatory infiltrate with lymphocytes and histiocytes, scattered eosinophils and rare neutrophils in interstitium	Cease implicated drug Corticosteroids	14

### Patient XTT–LTT results

Table 3 summarises the XTT–LTT results for the 8 DRESS and 2 AGEP patients tested as part of the initial validation. All patient data was analysed statistically by one way ANOVA or by calculating the stimulation index (SI) with a cut off, of > 2 considered positive. Ten patients were enrolled in the method validation. Eight patients had a clinical diagnosis of DRESS, and two had AGEP. Of the 8 patients with DRESS, the implicated drug was sulfasalazine (n = 2), Piperacillin/Tazobactam (Tazocin) (n = 2), allopurinol (n = 1), amoxicillin + clavulanic acid (n = 1), apixaban (n = 1), lenalidomide (n = 1) and vancomycin (n = 1). At least 150,000 cells were present in each well.

**Table 3:** XTT–LTT results for the 10 patients enrolled in the study

Patient	Implicated and culprit drugs	Cells per well	One way ANOVA	Stimulation Index (> 2 positive, < 2 negative)	Cytokines (pg/ml)		
					IL2	IL4	IL5
P1	Vancomycin	200,000	Vancomycin 100 µg/ml *	Vancomycin 100 µg/ml (2.57)	− ve	− ve	0.3
P2 †	Piperacillin/Tazobactam (Tazocin) Ceftriaxone (NEG) Ceftotaxime (NEG) Cefazolin (NEG) Ceftazidime (NEG) Amoxyicillin (NEG) Benzylpenicillin (NEG)	153.000	Piperacillin/Tazobactam (Tazocin) 100 µg/ml*	Piperacillin/Tazobactam (Tazocin) 100 µg/ml (2.33)	− ve	− ve	15–50
P3 †	Sulphasalazine Sulfapyridine Sulfamethoxazole Sulfamethoxazole + trimethoprim	200,000	Sulphasalazine (50 µg/ml***, 25 µg/ml* and 12.5 µg/ml*** Sulfapyridine 12.5 µg/ml*, 6.25 µg/ml** 3.12 µg/ml** Sulfamethoxazole 100 µg/ml**, 50 µg/ml*** Sulfamethoxazole + trimethoprim (NS)	Sulphasalazine 100 µg/ml (2.2) 50 µg/ml (3.3), 25 µg/ml (2.6), 12.5 µg/ml (3.3) Sulfapyridine12.5 µg/ml (2.55), 6.25 µg/ml (2.9), 3.12 µg/ml (2.97) Sulfamethoxazole 100 µg/ml (3.78), 50 µg/ml (2.66), 25 µg/ml (2.13) Sulfamethoxazole + trimethoprim 50 µg/ml (2.46), 25 µg/ml (2.0)	− ve	0.44	2–5
P4 †	Allopurinol Oxypurinol	200,000	Allopurinol 25 µg/ml* Oxypurinol 3.1 µg/ml*	Allopurinol 100 µg/ml (2.19) 25 µg/ml (2.63), 12.5 µg/ml (2.15)	− ve	− ve	− ve
P5 †	Sulphasalazine Sulfapyridine (NEG) Sulfamethoxazole (NEG) Sulfamethoxazole + trimethoprim (NEG)	170,000	Sulphasalazine (25 µg/ml*)	Sulphasalazine 25 µg/ml (4.97)	− ve	− ve	− ve
P6	Apixaban	200,000	Apixaban (25 µg/ml**,12.5 µg/ml**, 6.25 µg/ml and 3.1 µg/ml)	Apixaban All SI < 2	NT	NT	NT
P7 †	Piperacillin/Tazobactam (Tazocin)	186,000	Piperacillin/Tazobactam (Tazocin) (100 µg/ml*** and 6.25 µg/ml*)	Piperacillin/Tazobactam (Tazocin) 100 µg/ml (3.16) 6.25 µg/ml (2.45)	− ve	− ve	5–10
P8 †	Amoxicillin + clavulanic acid Amoxicillin (NEG)	200,000	Amoxicillin + clavulanic acid (NS)	Amoxicillin + clavulanic acid 25 µg/ml (2.43)	− ve	0.3–0.7	0.5–2.4 l
P9	Lenalidomide Allopurinol (NEG) Oxypurinol (NEG)	200,000	Lenalidomide (50 µg/ml* and 6.25 µg/ml*)	Lenalidomide SI < 2	− ve	NT	NT
P10	Benzylpenicillin Flucloxacillin Ceftazolin-AFT	200,000	Benzylpenicillin (NS) Flucloxacillin (NS) Ceftazolin-AFT 3.12 µg/ml*	Benzylpenicillin 12.5 µg/ml (3.75), 6.25 µg/ml (3.56) Flucloxacillin 25 µg/ml (4.03), 12,5 µg/ml (3.3), 6.25 µg/ml (3.45), 3.12 µg/ml (3.14) Ceftazolin-AFT 50 µg/ml (3.44), 12.5 µg/ml (2.5), 6.25 µg/ml (3.66), 3.12 µg/ml (4.02)	− ve	NT	NT

*NS* Not significant, *NT* = Not tested, *− ve/NEG* = Negative, *ANOVA* * p < 0.05, ** p < 0.01 and *** p < 0.001

SI greater > 2 positive

^†^Age and sex matched control with no history of reactivity to drug performed for this patient ^–^All were negative

**Fig. 3:**
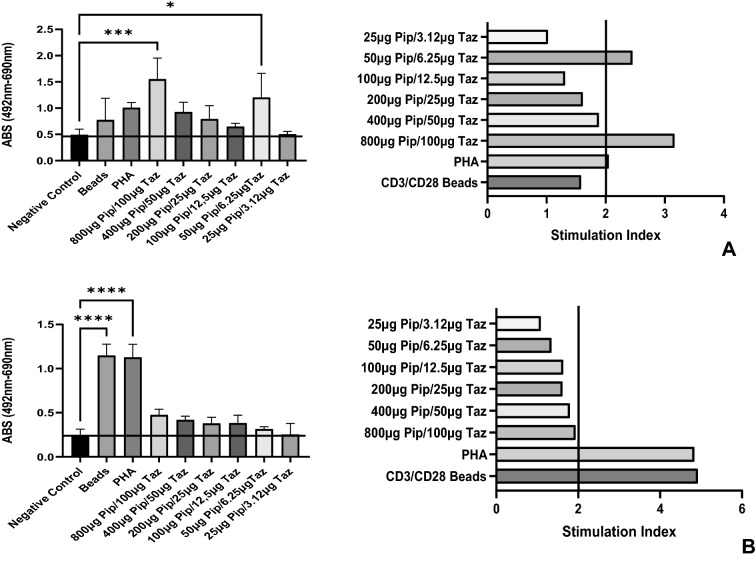
Patient 7 Analysis by ANOVA and Calculation of SI (**A**) and Age and sex matched control for patient 7 (**B**)

All 10 patients had a positive result using a combination of ANOVA and calculation of SI. Six patients, all with a diagnosis of DRESS, had positive results by both ANOVA and calculation of SI. Patient 6 with AGEP to apixaban, and patient 9 with DRESS to lenalidomide, had a positive result through ANOVA only. Patient 8 with DRESS to amoxicillin + clavulanic acid, and patient 10 with AGEP to benzylpenicillin had a positive SI only. The result of the ANOVA analysis and SI calculation for each patient are shown in Table 3. Figure 3 shows a graphical representation of results for patient 7 and their age and sex matched control.

All patients were negative for IL-2. Two patients had a low level of IL-4, and 5 out of 7 patients had some level of IL-5 secretion to the implicated drug. Two patients had no cytokine production and 3 patients were not tested.

### Real time XTT–LTT sample monitoring

All patients and controls had their XTT–LTT plates read at 4, 6 and 24 h time points before plates were frozen for cytokine analysis.

**Fig. 4:**
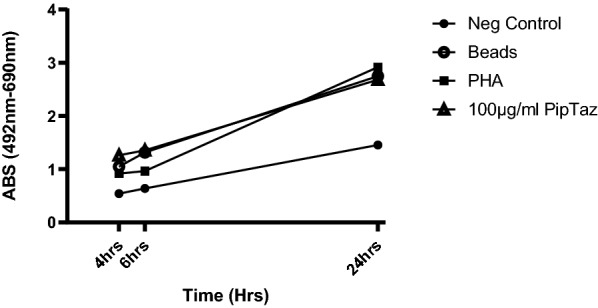
Real time monitoring of patient 7 samples over a 24 h period

Figure 4 shows the real time monitoring data for patient 7 over a 24 h period. Generally, the 4 h time detection time point is enough to show significant increased proliferation to culprit drug but later time points can be used to confirm results.

## Discussion

Our study focused on validating the XTT proliferation detection method for LTT for the diagnosis of patients with DRESS and AGEP. Over time, modifications to the classical 3H-thymidine LTT have been utilised to increase sensitivity, specificity and practicality. The classical LTT is based on the detection of drug-specific memory T cells in the peripheral blood of sensitized patients, and 3H-thymidine is added to the cell culture and incubated for 10–16 h, which is incorporated in the DNA of the proliferating cells. The amount of 3H-thymidine corresponds to the level of proliferation and is measured by detecting the radiation in counts per minute (cpm). In most cases a stimulation index (SI) is calculated which takes the biological variation into account. The SI value is calculated by dividing the cpm value of the drug-stimulated samples by the cpm value of the unstimulated sample. Generally, a SI value of ≥ 2 is considered a positive result, although a SI value ≥ 3 is considered positive for beta-lactams by some institutions [9].

Most data with 3H-thymidine-LTT show an overall sensitivity of 56% and specificity of 94%, respectively [10]. Higher results in 3H-thymidine LTT are dependent on the drug tested, and type of reaction, for example, higher readings are observed from anticonvulsant-DRESS than SJS/TEN [11]. False positive results can be generated with certain drugs such as methotrexate [12]. Interestingly, the performance data from 3H-thymidine LTT for severe reactions reveals reduced sensitivity; for severe bullous skin reactions 25–75% [13], with increased sensitivity for DRESS 67% [7]. In contrast to SJS/TEN, positive LTT reactions are obtained at the recovery phase i.e. 2 months after onset, but not during the acute phase of DRESS which may be due to activation of T-reg cells [14].

Modifications to classical 3H-thymidine LTT have been trialed to improve sensitivity. Removal of T-reg/CD + 25hi cells in a cohort of 11 patients with drug hypersensitivity resulted in an increase in sensitivity from 25 to 82.35% and an enhanced SI (2.05 to 6.02) [15]. In our study, depletion of T-regs did not increase signal to PHA and CD + 3/CD + 28 beads, however it reduced the background in the negative control (Fig. 2A) which could potentially improve sensitivity. Due to the associated addition processing time of an hour or more of cell sorting per patient and risk of contamination, we did not pursue this for the method evaluation.

The testing of reactive metabolites as well as the implicated inert drug appears important to avoid false negative results to certain drugs [16]. XTT–LTT results for patients 3 and 5 who had sulfasalazine-DRESS, showed that one was positive to the drug itself, whereas the other was only positive to the reactive metabolites, sulfapyridine and sulfamethoxazole. This demonstrates the importance of testing downstream metabolites of certain drugs [17, 18].

Classic LTT involves working with radioactive material (3H-thymidine), and most diagnostic laboratories are utilising less hazardous materials. Whilst neither the 3H-thymidine LTT nor our XTT–LTT can characterise the type of hypersensitivity reaction and proliferation is given per culture well and not per individual cell, no additional assays can be performed with or after 3H-thymidine incorporation [19]. Therefore, a distinct advantage of our XTT–LTT is the analysis of cytokine or even cells after reading. Further, a standard ELISA plate reader can be used for XTT–LTT instead of an expensive beta-scintillation counter. No dedicated radioactive handling facilities for use and disposal of contaminated radioactive waste is required. Finally, multiple time points can be read using the XTT–LTT method.

Alternative markers of lymphocyte proliferation were determined in this study. BrdU was more labour-intensive with the requirement for spinning cells down to fix. Moreover, this crucial fixing step often failed. We also found the sensitivity of the BrdU assay to be low as compared to the XTT–LTT assay. The CyQUANT™ assay was also more labour-intensive with the requirement for spinning cells down and removing the supernatant. The signal was weak after 60 min with only a slight increase at 3 h. The MTT-LTT assay is sensitive but can only be read at one time point. It requires addition of DMSO for reading to dissolve formazan crystals and thus no further analysis can be performed. The XTT–LTT assay in contrast was easy to perform by simply adding the reagent to each well without the need for spinning plates down or fixation. It can be read at multiple time points. We tested 4, 6 and 24 h time points (Fig. 4) and found that 4–6 h was optimum. Twenty-four, hour reading can be useful however in some patients to monitor specific samples and drug concentrations which have borderline significance at 4–6 h. The major advantage is that plates can be frozen after reading for further analysis, such as for cytokine measurement. We found that the XTT–LTT signal was sensitive enough to verify hypersensitivity for all patients in the study in contrast to other published data [20]. This study however was done in canines so XTT sensitivity could be vary in different species. The assay is robust enough to be performed following the acute phase of DRESS and in one patient up to 5 years following the reaction.

We initially tested drugs at concentrations ranging from 1 to 400 µg/ml but generally found concentrations > 100 µg/ml to be toxic and therefore dilutions from 100 µg to 3.12 µg/ml were used in this study. It is vitally important to minimise the occurrence of false negative results that a wide range of concentrations be tested as 40% of our cohort were only positive at one concentration (not dose dependent) demonstrating the complexity of drug/T cell interaction in vitro [21].

Evaluation of the addition of autologous serum showed a significant increase in the negative control which may have been due to the interaction of XTT with human serum albumin as found by other groups, and [22] thus was not included for the study patients.

Cytokine analysis of patient samples from XTT–LTT positive drug wells were negative for IL-2 for all patients as seen in other studies [21]. IL-4 was present at very low levels in patient 3 (0.44 pg/ml) and patient 8 (0.3–0.7 pg/ml) and was negative for all the 6 other tested patients. Other groups have suggested that IL-4 may be a useful additional marker for LTT, but this was only assessed in cases of phenytoin and carbamazepine-induced DRESS [23]. IL-5 was detected in 5/8 patients tested at a range of 0.3–50 pg/ml including both cases of piperacillin/tazobactam-DRESS. Interestingly, of the two sulfasalazine-DRESS patients, IL-5 was detected in patient 3 who reacted to both the drug and derivative but not in patient 5 who reacted to the drug only. The detection of IL-5 has thus far proven useful as a surrogate marker for hypersensitivity to metals only such as nickel and palladium [24].

The benefit of a LTT with high sensitivity in addition to specificity is for the evaluation of severe hypersensitivity reactions implicating more than one drug. Both allopurinol and lenalidomide were possible causes for DRESS in patient 9. Allopurinol is one of the leading causes of DRESS globally [25] with few cases associated with lenalidomide to date [26]. Surprisingly, the patient had a positive XTT–LTT to lenalidomide and not allopurinol or its metabolite oxypurinol [27]. The patient has consented to an allopurinol challenge if the drug is required in the future. In contrast, The XTT–LTT for patient 4 with allopurinol-DRESS was positive to both allopurinol and oxypurinol with no other agent considered clinically as a possible culprit. Confirmation of the likely causative drug as in our case(s) is useful in confirming the aetiology of the reaction and restriction of drugs with potential structural cross-reactivity.

The major limitation of this study is the small sample size, but it is method validation study that will now proceed to a clinical validation. Our cohort comprises mainly of patients with DRESS. The assay also performed well in both patients with AGEP indicating its potentially utility in this type of hypersensitivity reaction. The XTT–LTT requires evaluation in SJS/TEN patients, a cohort that traditionally has low sensitivity for LTT assays. The assay correctly diagnosed all 10 patients with varied drug culprits, including common precipitants of DRESS. Despite the robust performance of the assay, its optimal timing with serial measurements requires further elucidation.

In summary, our group has validated a LTT assay utilising XTT for detection of causal drugs in patients with DRESS and the method also holds promise for AGEP. The assay awaits a clinical validation study with an appropriate sample size and maybe sensitive enough to use following the acute phase of the DRESS reaction and for up to 5 years following the reaction.

## Data Availability

The datasets during and/or analysed during the current study available from the corresponding author on reasonable request.
